# Innovative and Cost-Efficient BiOI Immobilization Technique on Ceramic Paper—Total Coverage and High Photocatalytic Activity

**DOI:** 10.3390/nano10101959

**Published:** 2020-10-01

**Authors:** Zsolt Kása, Eszter Orbán, Zsolt Pap, Imre Ábrahám, Klára Magyari, Seema Garg, Klara Hernadi

**Affiliations:** 1 Department of Applied and Environmental Chemistry, University of Szeged, 6720 Szeged, Rerrich Béla sqr. 1, 6720 Szeged, Hungary; 2 Department of Organic Chemistry, University of Szeged, Dóm sqr. 8, 6720 Szeged, Hungary; eszterorban94@chem.u-szeged.hu; 3 Institute of Environmental Science and Technology, University of Szeged, Tisza Lajos blvd. 103, 6720 Szeged, Hungary; pzsolt@chem.u-szeged.hu (Z.P.); klara.magyari@ubbcluj.ro (K.M.); 4 Nanostructured Materials and Bio-Nano-Interfaces Center Interdisciplinary Research Institute on Bio-Nano-Sciences, Babes-Bolyai University, Treboniu Laurian Str. 42, 400271 Cluj-Napoca, Romania; 5 UniChem Ltd., Department of Development, T. 491, Kőiskola str. 3., 6760 Kistelek, Hungary; abrahamimre@unichem.hu; 6 Amity Institute of Applied Sciences, Amity University, Sector 125, Noida, Uttar Pradesh 201313, India; sgarg2@amity.edu

**Keywords:** bismuth oxyiodide, immobilization, photocatalysis, photocatalytic reactor, scale-up procedure

## Abstract

In the present work, visible light active bismuth oxyiodide (BiOI) was immobilized on a commercial, non-conductive support (an Al_2_O_3_ based ceramic paper) using a novel two-step spray coating technique and investigated with different characterization methods (e.g., SEM, Raman, XPS). Our main goal was to eliminate the separation costs after the photocatalytic measurement and investigate the chemical relevance and opportunity to use this technique in the industry. Our as-prepared uniform BiOI layer had similar properties to the well-known reference BiOI powder. The Raman and XPS measurements confirmed that the enriched amount of the surface iodine defined the color and as well the band gap of the BiOI layer. The durable BiOI layers have prominent photocatalytic activity under UV and visible light irradiation as well. The scale-up procedure proved that the designed BiOI coated paper was reusable and potentially applicable in the industry by straightforward scale-up, which is due to the elaborated non-conventional BiOI coverage estimation method. This immobilization technique could open several opportunities for immobilizing many other visible light active photocatalysts with simple materials and low cost.

## 1. Introduction

Photocatalysis has progressed significantly as a water treatment method and reached such a technological level of expansion that developing countries can afford their application for water treatment, because nowadays the applied semiconductors possess sufficiently high degradation yields, and low production cost. One of the most impedimental factors of photocatalytic industrial water treatment is the immobilization of the semiconductor to eliminate the separation/filtration costs [1]. In most cases, UV active titanium dioxide was used in photocatalytic water purification applications and on the semi-industrial scale (both immobilized and in suspension as well) [2,3,4,5]. This material has some advantages and disadvantages [6], but it is much more critical to solve the immobilization related problems. Moreover, if visible light active catalysts are needed to be immobilized, their deficiencies during immobilization should be avoided.

Therefore, a semiconductor which is excitable under visible light as well is needed, considering that a significant part of the emitted sunlight is also in the visible region (~44%), while only a small fraction is in the UV light region (3–5%) [7]. Several visible light active photocatalysts are accessible; nevertheless, the chosen semiconductor must have appropriate physical and chemical properties, like titanium dioxide, such as photostability, chemical, and biological inertness, etc. [8]. In recent decades, the scientific publications focused on visible light active photocatalysts’ potential, and successfully increased the degradation efficiency of many photocatalysts, such as WO_3_ [9], Ag_3_PO_4_ [10], or bismuth oxide-based materials, namely bismuth tungstate [11,12,13], bismuth vanadate [14], bismuth molybdate [15], or bismuth oxyhalides [16,17,18].

The bismuth oxyhalides (BiOX; X=Br, Cl, I, F) are a new group of visible-light-driven photocatalysts with a layered structure, where halogen ions are between the (Bi_2_O_2_)^2+^ layers and interconnected by electrostatic forces, which yields a plate-like morphology. The unique morphology could induce more effective separation of the photoinduced electron-hole pairs [19,20,21]. One member of the BiOX is the bismuth oxyiodide, which has the narrowest band gap (≈1.91 eV), compared to the others, and it has similar good physico–chemical and mechanical properties besides high chemical and optical stability, corrosion resistance, and nontoxicity [22,23,24]. Consequently, the BiOI is a potentially useful alternative semiconductor, which is excitable by visible light, and sufficiently stable to be used in water purification, and it can degrade some organic pollutants such as methyl orange, or methylene blue [25]. Most of the studies conducted with BiOI photocatalysts were in suspensions [26,27], just a few papers paid attention to the immobilization possibility [28,29,30,31], but the experiments were conducted only on a lab scale. Unfortunately, to the best of our knowledge, the literature is not dealing with any information on successfully and low-cost immobilization techniques with a sufficiently large surface to become a relevant essential component for the alternative water purification technology [32], so the next notable milestone of the development is elaborating the appropriate immobilization BiOI technique.

Two main directions can be distinguished in the applied immobilization procedures. The first one is trying to fix the crystallized photocatalysts on a surface using materials with adhesive properties, such as organic or inorganic glues (polyvinyl alcohol, a hydrolyzed amorphous titanium dioxide of polymer glue) [33,34,35]. This immobilization method may have some risks; namely, that the photocatalyst degrades the glue, while the glue can poison or cover the photocatalyst [36], reducing the degradation efficiency [34]. Furthermore, the fixing material should inhibit the degree of photocatalyst leaching during the water purification [37]. The other pathway is trying to create a unified photocatalyst layer on any surface like a paint on a metal surface, thereby preserving the properties of the catalyst while minimizing the leaching [38]. The biggest advantage of this technique is that the photoactive layer could be stable until the photocatalyst layer not physically damaged. Furthermore, it is not necessary to create physical or chemical bonding between the support and the photocatalysts [39].

The last step is to choose the support for BiOI, which must be photocatalytically inert, cheap, undemanding, and resistant. A potentially suitable carrier could be alumina or silica [40], carbon fiber [41], glass fiber, PET, etc. [42]. Veréb and co-workers used alumina (α-Al_2_O_3_) based non-woven ceramic paper for their TiO_2_ support and proved that the ceramic paper is potentially adaptable into industry [43]. Furthermore, the alumina-based ceramic paper was highlighted by its excellent size and shape capacity, flexibility, electric properties, thermal stability, chemical resistance, and not least extremely low price (10–20 USD/m^2^) among the other available supports [31].

In this paper, shape-controlled BiOI microparticles were immobilized in situ on ceramic paper by two different fixing processes to eliminate the separation step after the photocatalytic reaction, thereby facilitating the reuse of the BiOI. A one-step hydrothermal method and a brand-new spray-coating technique were elaborated, which have the potential to be scaled up to industrial scale. The photocatalytic activity was investigated in a unique flow reactor, and the photocatalytic tests result in a relationship with the coated layer and the coverage, which was highly dependent on the applied solvent. A clear correlation was found between the band gap and the two main crystal plane ratios. Furthermore, the spray coating technique was successfully scaled up to the pilot-plant level as well.

## 2. Experimental

### 2.1. Materials

All reagents were of analytical grade and used without any additional purification. During the immobilization Bi(NO_3_)_3_⋅5H_2_O (Sigma-Aldrich Ltd. Hungary), and potassium iodide (KI) (VWR Int. Ltd. Hungary) were used as precursors, while the applied solvent was ethylene glycol (VWR Int. Ltd. Hungary), and the “transport medium” was ultrapure Milli-Q water, absolute ethanol (VWR Int. Ltd. Hungary), or isopropanol (VWR Int. Ltd. Hungary). The support material was a commercial insulator, an 8 × 20 cm non-woven alumina-based paper sheet (COTRONICS Co., USA, NY, 1.6 mm thickness Catalog No.: 300-040-1).

### 2.2. Direct In-Situ Immobilization by Solvothermal Synthesis

During a typical synthesis, 2.073 g bismuth nitrate pentahydrate was dissolved in 200 mL ethylene glycol (EG). The solution was stirred at 50 °C for 30 min, then 0.711 g KI powder was added to the clear solution, under vigorous stirring, followed by an additional 30 min homogenization (the molar ratio was 1:1). The yellowish solution was transferred into a paint-roller, which was equipped with two slightly rough-surfaced rollers. The ceramic paper was rolled between these cylinders several times, to impregnate the “catalyst ink” into the alumina fibers. During this step, a water-free environment and tools were assured to prevent the hydrolysis of the “catalyst ink”. The impregnated ceramic paper was transferred into a 210 mL Teflon-lined stainless-steel autoclave, and the remaining “catalyst ink” was also transferred into the autoclave. The crystallization temperature was set to 120 °C for 3 h, while the cooling was at room temperature, naturally without forced air convection. The ceramic paper’s color changed to orange, demonstrating the first evidence of the formation of BiOI. The alumina paper samples were transferred into a beaker on a magnetic stirrer, which assisted the cleaning procedure with absolute ethanol and Milli-Q water 3-3 times, while the crystallized powder was also washed and centrifuged with absolute ethanol and Milli-Q water also 3-3 times. The powder and the coated ceramic paper were dried at 40 °C for 24 h. The mass of immobilized BiOI was checked by weight measurement. The abbreviation of the BiOI coated paper was “CP_EG_h.t.”, while the remaining BiOI powder was named as “P_EG_h.t.” (specific surface area: S_BET_: 28.7 m^2^/g).

### 2.3. The Spray-Coating Immobilization Technique

The second immobilization technique was different from solvothermal synthesis. It should be noted that this process was chosen to form a BiOI layer on the surface of the ceramic paper, when the pilot-plant scaled experiment was carried out. The typical procedure was as follows:

First, 1.037 g bismuth nitrate pentahydrate was dissolved in 50 mL solvent (Milli-Q water, isopropanol, absolute ethanol) or solvent mixture (50 *w/w*% ethanol and 50 *w/w*% Milli-Q water or 50 *w/w*% ethylene glycol and 50 *w/w*% absolute ethanol) with different composition with a magnetic stirrer or suspended by ultrasonication combined with stirring. After that, 0.356 g KI was added to the solution or suspension (the molar ratio was 1:1), then the solution/suspension became orange–yellowish, which was the catalyst ink. This was transferred into a tank and sprayed on the ceramic paper surface using nitrogen gas flow. After the spray-coating process, the deposited ink was gradually blackish in color which was identified as BiI_3_ layer by XRD [29]. To evaporate all the solvents, the ceramic paper was dried at 40 °C for 24 h. The washing step was repeated three times with absolute ethanol and three times with Milli-Q water, while an orange or yellowish layer formed. The amount of the immobilized BiOI was checked by weight measurements (Table 1).

The obtained samples were coded using the applied solvents, as follows:

- CP_MQ_s.c.: Milli-Q water,
- CP_EtOH_s.c.: Absolute ethanol,
- CP_i-Pr_s.c.: Isopropanol,
- CP_EtOH+MQ_s.c.: Absolute ethanol and water mixture (50–50 *w/w*%),
- CP_EtOH+EG_s.c.: Absolute ethanol and ethylene glycol mixture (50–50 *w/w*%).

**Scheme 1:**
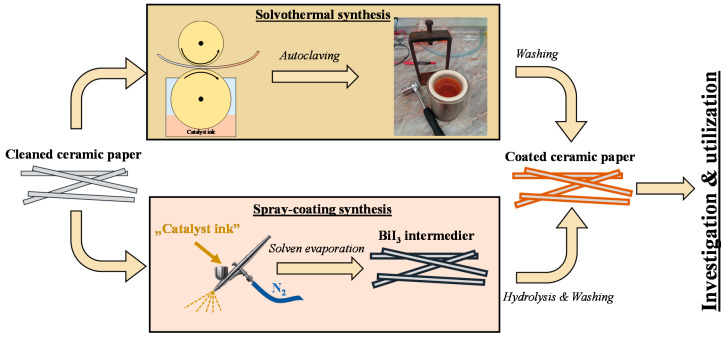
Different bismuth oxyiodide (BiOI) deposition methods. Top: one-step solvothermal method in ethylene glycol media; bottom: two-step spray-coating method using different solvents (Milli-Q, absolute ethanol, isopropanol or mixture of these solvents).

The “CP” coded the ceramic paper, while the “s.c.” implies the spray coating technique. The scheme of the different preparation methods is shown in Scheme 1.

### 2.4. Characterization Methods

The BiOI coated ceramic papers, and the powder were characterized by a Rigaku MiniFlex Type II X-ray diffractometer (Japan) with CuKα radiation (λ = 0.15406 nm). The diffractograms were recorded in a 2θ° range from 5° to 65°, with a speed of 3°·min^−1^. The Scherrer equation was applied to evaluate the primary crystallite size. The morphology of the particles was analyzed by a Hitachi S-4700 Type II (Japan) cold field-emission scanning electron microscope (SEM), operating at 10 kV, equipped with an EDX Röntec QX2 spectrometer (Germany) with Be window. A WVR-SZT250 optical microscope (VWR Ltd. Hungary) equipped with a VisiScope CB-5 camera (VWR Ltd. Hungary) was used to estimate the efficiency of the coverage. A total of 20 to 30 pictures were taken in 7-fold, and 45-fold zoom with 2592 × 1944 resolution, and these pictures were analyzed separately by ImageJ software.

A JASCO-V650 UV–Vis spectrophotometer (Japan) with an integration sphere (ILV-724) was used to record the diffuse reflectance spectra of the coated ceramic paper and BiOI powder in a 250–800 nm wavelength range. The DR-spectra were differentiated in the function of the wavelength (dR/dλ). The first-order derivations were performed between 400 nm and 750 nm to evaluate the possible electron-transition bands. Furthermore, the classical Tauc-plot was used, and the bandgap values were calculated by the Kubelka–Munk equation. The infrared spectra were recorded using a Jasco IRT-5000 FT-IR microscope (Japan) coupled to a Jasco FT-IR-6200 spectrometer (Japan) in reflection configuration at room temperature, in the range of 700–4000 cm^−1^; spectral resolution 4 cm^−1^. In the case of the BiOI and intermediate powders analysis, the well-known KBr pellet technique was applied.

Thermo Scientific DXR Raman microscope (USA, MA.) was used to obtain information about the crystallographic environment. The light source was a diode-pumped, frequency-doubled Nd:YAG laser, which was used with a 15 mW maximum laser power (λ = 780 nm). The spectra were recorded between 50 cm^−1^ to 3500 cm^−1^ with 2 cm^−1^ resolution at room temperature. The Fourier-Transformed self-deconvolution method was used in order to identify the overlapped curves and locate the peak positions. The oxidation states of the elements in BiOI layer were recorded with a Specs Phoibos 150 MCD photoelectron spectroscope device (Germany) using monochromatized Al Kα radiation (1486.7 eV) at 15 kV and 20 mA. The pressure was lower than 10^−9^ bar. The resolution of the Bi4f and the I3d spectra were 0.05 eV. Before the measurement, top of the coated paper was removed with a scalpel in order to minimize the sample charging and electron scattering.

### 2.5. Photocatalytic Activity Measurements

The photocatalytic activity was investigated in a unique bath reactor. The coated paper size was 6.6 × 15 cm (~0.01 m^2^), which was locked on the flowing channel slope (10°) of the flow reactor by two stainless steel rods. The buffer reservoir was thermostated at 25 °C and contained a magnetic stirrer to provide homogenization. A centrifugal pump (3.5 L/min) was responsible in assuring the recirculation of the rhodamine B solution (initial concentration: 0.01 M, V_RhB_ = 0.5 L). A uniform liquid film was assured on the coated ceramic paper surface by a low cascade type of liquid distributor.

A total of 2 40 W UV and 2 × 40 W visible light fluorescent lamps were used as a light source above the bath (irradiation height = 10 cm). In the case of the visible light tests, a UV filter foil was applied to exclude any incident UV photons. Before the lamp switched on, the RhB solution was circulated in the dark for 30 min. In order to stabilize the adsorption–desorption equilibrium, the reactor was covered by aluminum foil.

After the lamps were switched on, 2 mL RhB solution was collected every 30 min and the RhB concentration was measured by UV–Vis spectrophotometry (detection wavelength: 553 nm). The concentration values were corrected, considering the evaporation of the water during the experiment. The photocatalytic tests were repeated three times, while the degree of leaching was also checked by weight measurement.

## 3. Results

### 3.1. X-ray Diffraction of Anchored BiOI

All coated ceramic paper showed diffraction peaks corresponding to BiOI (JCPDS card number: 10-0455). The following major diffraction peaks were identified: (102), (110), (111), (112), (004), (200), and (212). The peak intensity values and peak ratios varied with the different spray coating methods applied or solvothermal solvent used. The ceramic paper support was not detectable (Figure S1).

**Figure 1:**
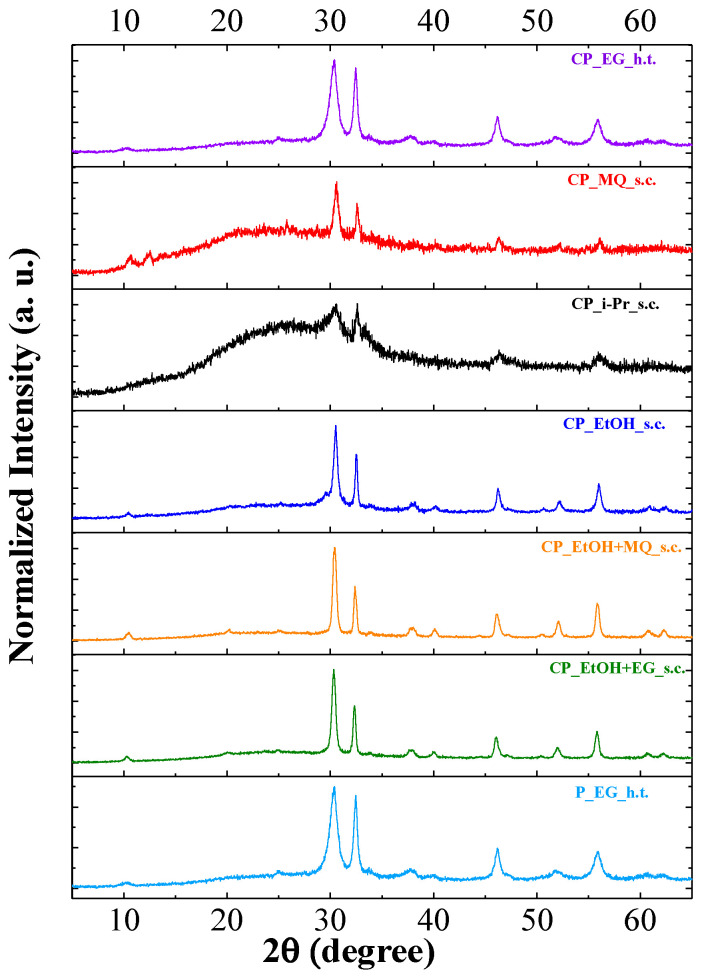
X-ray diffraction pattern of the BiOI layer on the ceramic paper: the effect of the solvent or the solvent mixture on the crystallographic properties and the two main peak ratios (*I*_(102)_*/I*_(110)_).

The XRD pattern (Figure 1) of the samples demonstrated that BiOI with similar crystallographic properties could be obtained on the surface of the ceramic paper by spray-coating at room temperature, as in the case of the solvothermal method, for maybe the first time in the literature.

The average primary crystallite size of BiOI was calculated using the Scherrer equation, alongside the mass of the deposited photocatalyst (Table 1). In the case of the solvothermal synthesis, the immobilized and the non-fixed powder catalyst showed nearly identical primary crystallite sizes (11 nm and 12 nm). The average crystallite size of the other samples (CP_MQ_s.c., CP_EtOH+MQ_s.c., and the CP_EtOH+EG_s.c.) was 21 nm, except the one prepared in the presence of isopropanol assisted spray-coating (CP_i-Pr_s.c.: 11 nm), which has the least intense reflection. The total mass of the BiOI achieved a maximum of 10% (between 50 mg and 440 mg) from the weight of the ceramic paper.

### 3.2. DRS—Diffuse Reflectance Spectroscopy

**Figure 2:**
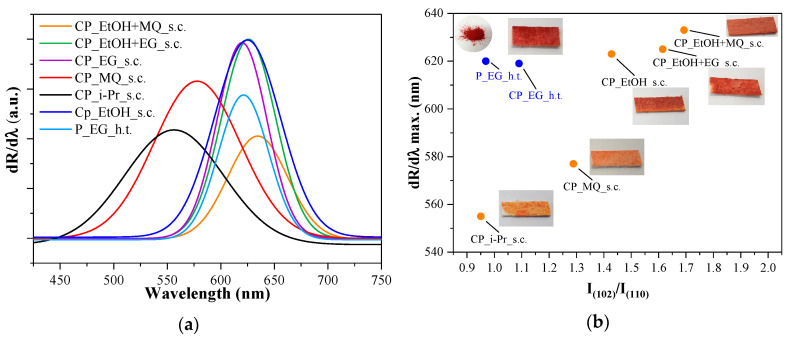
Measured optical properties and photos of immobilized BiOI catalysts (**a**): first derivative of diffuse reflectance spectra (**b**): correlation between the dR/dλ maximum and the ratio of the two main crystallographic planes.

The color of the coated papers was different for each sample; thus, the optical properties were investigated by DRS measurement. All coated papers’ (and the powder from the solvothermal synthesis) bandgaps varied between 1.90 to 2.13 eV, which means that all samples are potentially excitable under visible light. Furthermore, the first-order derivation was applied to obtain more information on the optical properties of the BiOI layer (Figure 2a). The light absorption maxima were located between 555 nm to 633 nm, which corresponded with the calculated bandgap. However, two exceptions were noted, namely samples CP_MQ_s.c. and CP_i-Pr_s.c., which showed their derived maximum values at 576 nm and 554 nm, respectively, totally different from the others. Interestingly, these two materials also showed lower diffraction peak intensity values than the other samples. Based on this, the DRS data (dR/dλ maxima) and the ratio of the I_(102)_/I_(110)_ were investigated closely (Figure 2b).

The crystallographic plane intensity ratio of (102) and (110) varied between 0.95 and 1.71, and this ratio followed the dR/dλ maxima position in the case of the samples obtained by the spray coating method. Considering that the dR/dλ is directly related to the color of the coated CP, consequently, the color of the samples also follows the intensity ratio of the already mentioned crystallographic planes. When the intensity of the (110) plane approaches the (102) intensity, the color of the BiOI exhibits a blue shift. A possible reason for the DRS–XRD correlation is that alongside the (102) crystal plane the concentration of the surface I‾ ions are higher than alongside the (110) [44].

### 3.3. SEM and Optical Microscope Investigation

The micromorphological features were investigated by scanning electron microscopy to obtain more information about the anchored BiOI layers. However, the high amplification and the monochromatic imaging of SEM did not allow to application of this technique to evaluate the degree of ceramic paper coverage. For this reason, in this section, the SEM images and the optical microscope images were evaluated together, using the “ImageJ” software.

**Figure 3:**
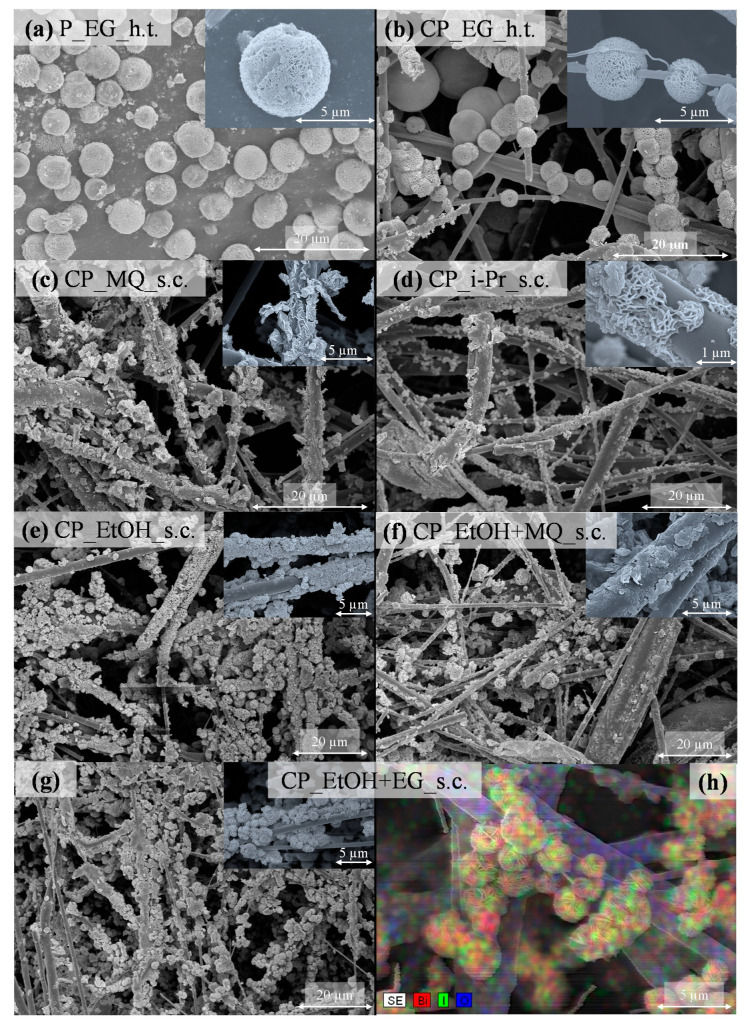
Effects of the solvent/matrix on the BiOI morphology, (**a**): well-structured BiOI spheres reference, (**b**): crystalized BiOI on the ceramic paper by solvothermal method. BiOI layers on the ceramic paper using different solvent: (**c**): milli-Q water, (**d**): isopropanol, (**e**): absolute ethanol, (**f**): absolute ethanol, and Milli-Q water mixture, (**g**): absolute ethanol and ethylene glycol mixture. (**h**): The distribution of elements.

After the solvothermal crystallization method, well-known and well-defined bismuth oxyiodide structures were formed, which were built up by individual plates and aggregated sphere-like structures with a 5 µm average diameter (Figure 3a). This type of material organization was also observable when the CP was applied during the solvothermal synthesis (Figure 3b), but the diameter of the hierarchical spheres varied from 2 µm to 10 µm. Interestingly, the crystalized BiOI plates grew around the CP fibers. Based on SEM micrographs, the BiOI coverage is not perfect, even though the photographs showed the opposite.

The papers obtained by spray-coating achieved almost total coverage, but the morphology of the BiOI was slightly differed. The primary plate-like structure also appeared, but did not assemble into spheres, rather it formed a unified layer (Figure 3c–g) on the alumina fibers.

When the water or the water/ethanol was the solvent (Figure 3c,f), poorly specified structures were formed, where the well-defined BiOI plates were not recognizable, instead it seems that a uniform monolayer formed. The EtOH and EtOH+EG solvent mixtures (Figure 3d,e) were caused by the characteristic plate-like morphology of BiOI with closely similar coverage. These findings may suggest that the alcoholic solvent or the mixture of the solvents which contained alcohol may result in better-defined morphology, while the water component may sinter the plates. Based on these results, by increasing the carbon chain of the alcohol, the isopropanol assisted BiOI layer (Figure 3g) approached perfect morphology with well-separated plates, while the coverage is not perfect.

The BiOI layer thickness was also measured, based on the SEM micrographs, which was estimated to be 0.16−2.35 µm sized. Unfortunately, there is no clear correlation between the thickness and the amount of fixed BiOI. The SEM–EDX mapping figure of CP_EtOH+EG_s.c. (Figure 3h) represents the element distribution of BiOI layers on the ceramic paper, while the atomic ratio of all samples is listed in Table 2. With good approximation, the ratios of the iodine to bismuth were one to one without significant elemental segregation in case of the powder and the BiOI layers, although somewhere tiny bismuth segregations were detected.

**Figure 4:**
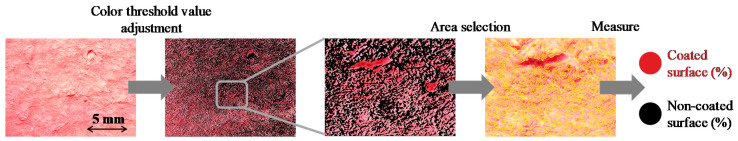
Surface coverage estimation process by the evaluation of the optical microscope images step by step using ImageJ software.

In order to obtain an overall picture concerning the coverage, an optical microscope was used, and the taken pictures were analyzed by ImageJ. The software calculated the ratio of the white and non-white areas, as can be seen step by step in Figure 4. These values were averaged and presented, including both magnifications. The surface coverage ratio (Table 1.) was found to be between 59.3% and 99.6%, which means that not all the investigated spray-coated techniques resulted in perfect coverage. Consequently, the coverage ratio values are considered when the photocatalytic activity measurements are analyzed.

**Figure 5:**
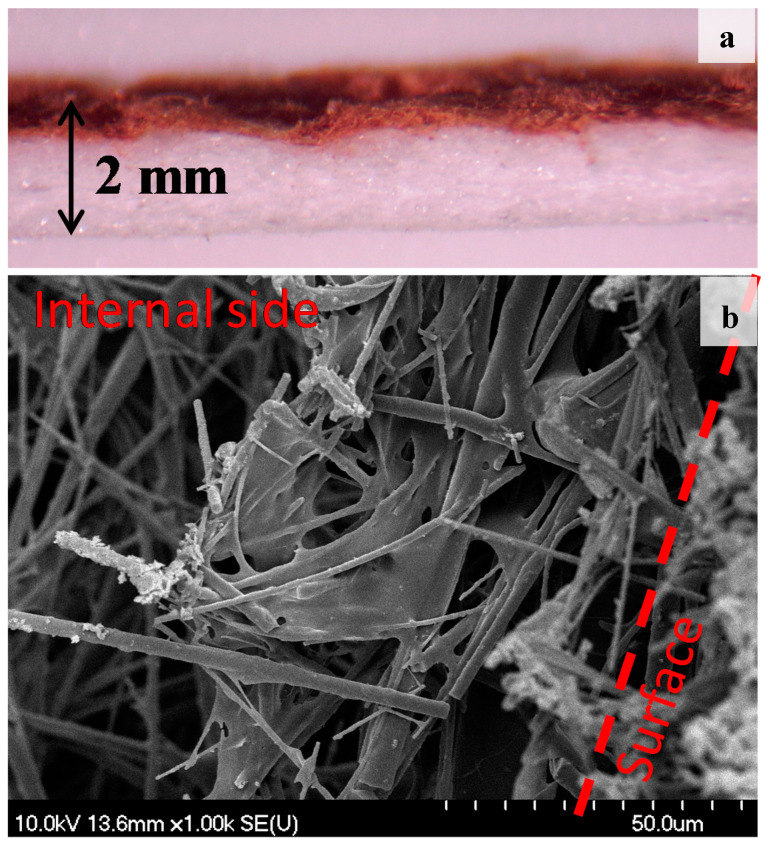
Distribution of the immobilized BiOI: Vertical optical image (**a**) and SEM micrograph (**b**) of the CP_MQ_s.c. two-step spray-coated ceramic paper.

After examining the surface of the coated papers, 5–10 pictures were taken alongside the tilted ceramic paper to obtain information about the thickness of the BiOI layer. The homogenous layer thickness was between 0.21 and 0.25 mm, while isolated BiOI regions were rarely detected inside the ceramic paper, which was confirmed by SEM and is shown in Figure 5. It means that the “catalyst ink” penetrating into the pores of the ceramic paper was low, which is entirely possible because the CP wettability is low; thus, not all the immobilized catalyst particles could be active during the photocatalytic process.

Furthermore, the BiOI distribution on the surface and in-depth was acceptable in the coated part of the CP (the top 10% of CP). The standard deviation was less than 3%.

### 3.4. Infrared Measurements

**Figure 6:**
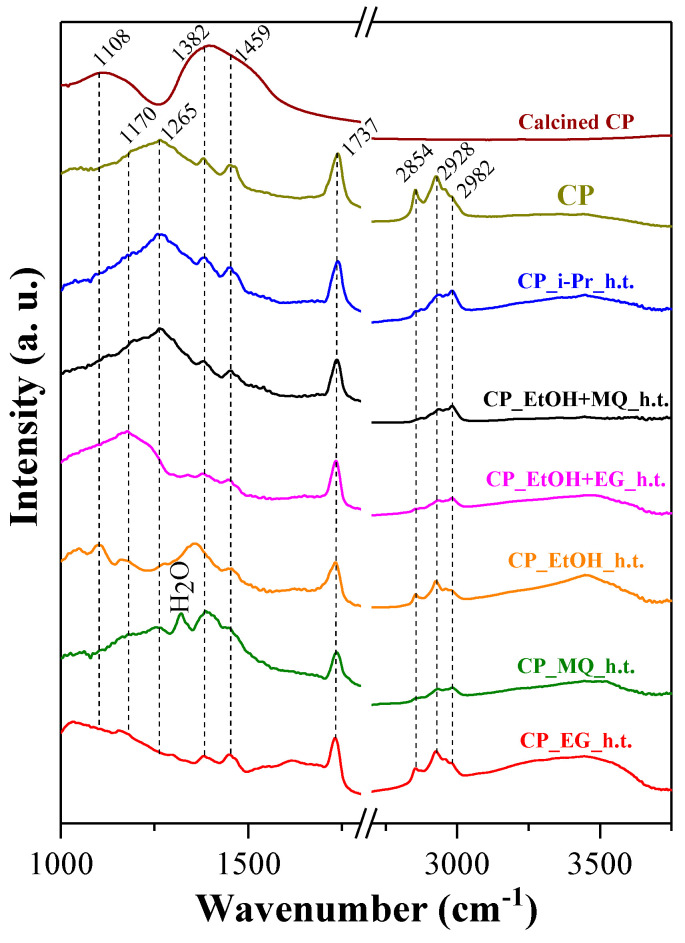
Infrared spectra of the BiOI coated papers marked the primary bindings of the ceramic paper and the epoxy resin. The different solvents and the solvent mixtures have no significant effects on the signals of the epoxy resin.

Infrared spectroscopy was used to investigate the anchoring of BiOI particles on the ceramic paper surface. Due to the rugged ceramic paper surface and the applied measurement methodology, the recorded IR spectra under 1000 cm^−1^ showed a very high noise/signal ratio, which means that the characteristic BiOI bonds were not detectable [45]. Hence, only indirect information can be obtained from the IR spectra in Figure 6.

It should be noted that the catalyst support was alumina-based ceramic paper; however, it also contained Si, and the ceramic structure was linked by an epoxy resin. Consequently, the following bonds were identified: at 1108 cm^−1^ an Si-O-Si asymmetric stretching bond was detected, while two bands at 1382 cm^−1^ and 1459 cm^−1^ were assigned to the Al-OH asymmetric stretching [46]. The intensity ratio and position of these signals did not change compared to the bulk ceramic paper, which may indicate that the BiOI did not bind through the Si-O units. Unfortunately, the typical bonds of Al-X were not detected clearly because these signals are under 1000 cm^−1^ [47].

Simultaneously, the CH_n_ stretching vibration bands were detected at 2854 cm^−1^, 2928 cm^−1^, and 2982 cm^−1^, respectively, and three other signals at 1170 cm^−1^, 1265 cm^−1^, and 1737 cm^−1^ [48], which could be assigned to the organic C-O bonds from epoxy resin. Interestingly, these bands disappeared after the calcination procedure. Furthermore, wide absorption bands of OH stretching groups appeared between 3300 cm^−1^ and 3500 cm^−1^, which originate from the BiOI [45].

The position and intensity of the C–O bond at 1737 cm^−1^ from the epoxy resin were also constant throughout the samples. If we consider that the C-O bond frequently as an electron donor (Lewis base), along with the Bi^3+^ of BiOI as electron acceptor (Lewis acid) [49], after the synthesis, additional Bi-O could be formed (where the O atom originated from the epoxy resin), which should have been visible in a shift of the C-O band position. This did not occur; therefore, it means that BiOI bound to the Al-O unit directly or a uniform BiOI layer formed without any chemical interaction.

### 3.5. Raman Measurements

According to the Schoenflies classification, bismuth oxyiodide has a D_4h_^7^ symmetry, which means that the BiOI is a Raman active material. The Raman spectra were recorded to gain more information about the BiOI coating.

**Figure 7:**
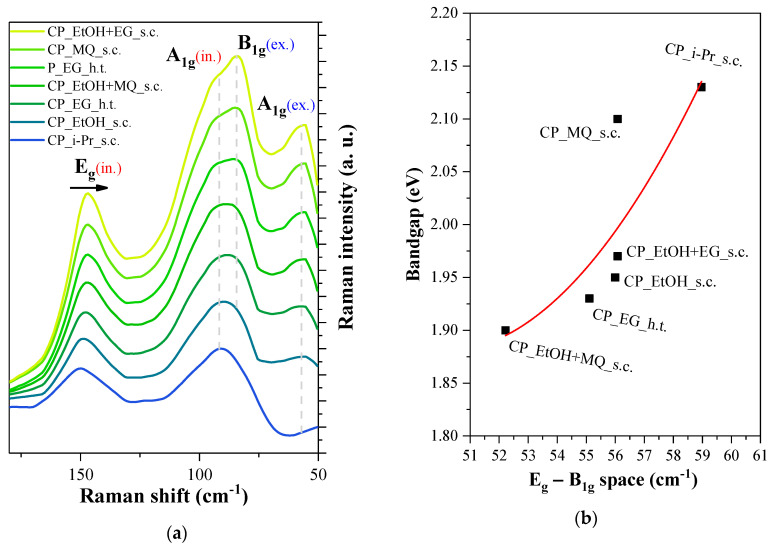
Raman spectra of the coated paper: the relationship between internal (A_1g_) and the external (B_1g_) Bi-I bond (**a**), and its effect on the optical properties and the calculated bandgap (**b**).

Two major characteristic peaks were detected, which were attributed to the A_1g_ and E_g_ Bi-I internal stretching mode at 90.0 cm^−1^ and around 148 cm^−1^ Raman shift, respectively [50]. Interestingly, the E_g_ Raman peak shifted to the lower region (from 149.7 cm^−1^ to 143.1 cm^−1^). All Raman spectra from 55 cm^−1^ to 130 cm^−1^ were deconvoluted (Figure S2), located and identified the overlapped peak. After the deconvolution, two new peaks were identified at 84.4 cm^−1^ and 60 cm^−1^ (std. dev: ±0.5 cm^−1^), which were assigned to the Bi-I external B_1g_ and external A_1g_ stretching bond [51,52,53]. These changes occurred together, which may signify that the crystallographic environment has slightly changed, but the main properties remained, therefore, the internal–external Bi-I relation was inverted (Figure 7a). The properties’ consistency was also proven by the strictly constant ratio of the Raman peaks’ intensity of E_g_ and the A_1g_/B_1g_ mode (0.57 to 0.62).

The E_g_ peak shifting and the B_1g_ peak intensity growth were found to be interconnected, which means that the internal Bi-I bonds continuously transformed into external Bi-I bonds, which means that the iodine should be enriched near the surface, and less Bi-O bonds can be found on the surface. This internal–external bond reversal is discernible on the bandgap, from which it was assumed that the amount of surface iodide was increased (Figure 7b).

### 3.6. XPS Measurements

**Figure 8:**
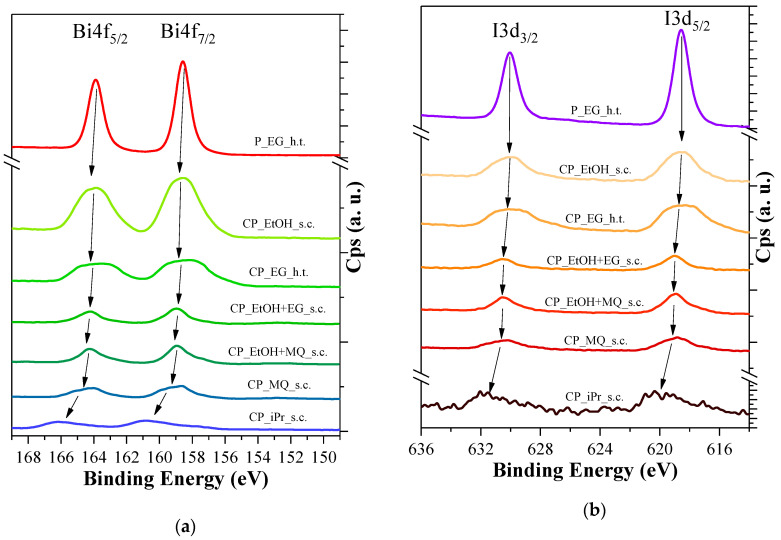
Bi4f spectra (**a**) and the I3d (**b**) XPS spectra of the immobilized BiOI layer. The shift of the peak position due to the different solvents and the solvent mixtures indicated the change of the surface chemical environment.

The surface chemical composition and the oxidation states of the elements in the BiOI layers were investigated by XPS. Due to the amorphous character of the ceramic paper the quantitative analysis was not possible; thus, a semiquantitative approach was applied. In the case of Bi4f spectra, two characteristic peaks were detectable at 158.6 eV (Bi4f_7/2_) and 163.9 eV (Bi4f_5/2_), which shifted to higher binding energy (160.9 and 166.2 eV), while the peak intensity decreased as well (Figure 8a). This could be due to the replacement of Bi^3+^ and I^−^ interactions with Bi^3+^ and O^2−^ [54] because Bi-O bonds are stronger than Bi-I.

At the same time, I3d spectra were also recorded (Figure 8b). Two characteristic peaks were identified at 618.6 and 630 eV, which were attributed to the I3d_5/2_ and I3d_3/2_, respectively. In case of the coated ceramic paper, the peaks were shifted toward higher binding energy values (620.1 eV and 631.7 eV) and decreased in intensity with the same trend. This peak movement could indicate the appearance of iodine vacancies [55], which may be direct evidence that the surface I‾, more precisely the amount of the external Bi-I binding, decreased, which proves that the surface iodide could define the optical properties and the band gap of the BiOI photocatalyst layer.

**Figure 9:**
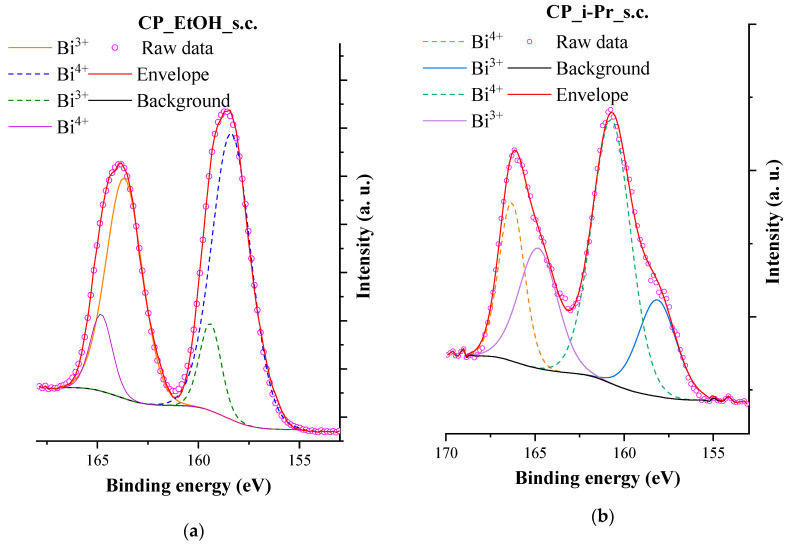
Deconvoluted Bi4f spectra of the CP_EtOH_s.c. (**a**) and the CP_i-Pr_s.c. (**b**), and the identified oxidation states of Bi.

The Bi4f spectra were deconvoluted to identify the oxidation states of bismuth (Figure 9a). In the case of the CP_EtOH_s.c. two major peaks were detected at 158.4 eV and 163.6 eV, which could be attributed to the Bi3+, while two minor peaks were discovered at 159.4 eV and 164.9 eV, which could be attributed to the Bi^4+^ [56]. This doublet existed in all cases. The trivalent bismuth presence was expected; namely, the Bi(III) is the most stable and most common oxidation state in the case of bismuth containing oxides [12,17,22]. The presence of the Bi(IV) can be a good sign of outstanding photocatalytic activity. Furthermore, the isopropanol solvent caused the most conspicuous changes. The Bi4f spectra shifted to higher energy (centered at 160.6 eV and at 166.3 eV), and the intensity ratio was reversed, which means that the Bi^4+^ was dominating in the case of CP_i-Pr_s.c. (Figure 9b).

### 3.7. Photocatalytic Activity Measurements and Different Approaches to the Activity

The photocatalytic activity was investigated under UV and visible light irradiation in a flow reactor. The photocatalytic activity of the powder was also investigated, but in the millimolar concentration range; the RhB adsorption was too high to be comparable with the immobilized BiOI.

**Figure 10:**
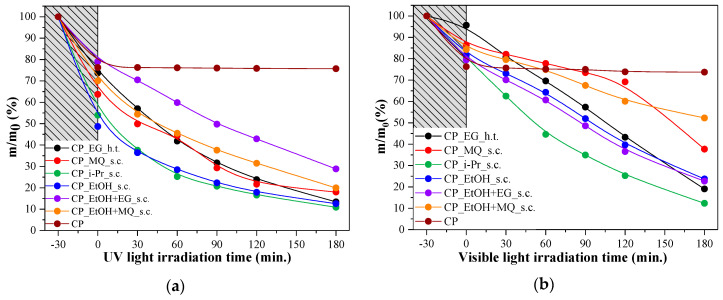
Rhodamine B photocatalytic degradation measurements under UV (**a**), and visible light irradiation (**b**) with an immobilized BiOI layer in a unique flow reactor.

During the tests, the evaporation rate was 5–7% after 3 h, although the buffer tank was thermostated; thus, an evaporation correction was applied, and the m/m_0_ values are presented in the photocatalytic degradation curves. The -ln(C/C_0_) was also plotted against time (Figure S3), and the reaction rate constant (k) was calculated. The non-coated paper was also tested, and it had no activity under neither UV nor visible light irradiation; only ≈20% RhB adsorption was noted. First, the UV activity was measured; after that, the papers were washed and dried, and then the activity of the same paper was tested under visible light irradiation. As Figure 10. shows, the adsorption rate decreased before the visible light tests, which may be due to the RhB molecules which were not removed during the cleaning procedure. In most cases, relatively, the same photocatalytic activities were experienced under visible light irradiation as under UV.

The best sample was CP_i-Pr_s.c. paper, which eliminated 90% of the mass of dye molecules after a 3-hour treatment. At the same time, it should be considered that the total amount of the degraded RhB is not the most accurate approach due to the differences in the BiOI layer properties (e.g., coverage, amount of the fixed BiOI).

If we take into consideration the amount of the immobilized BiOI (Table 1) and recalculate the degraded RhB, sample CP_MQ_s.c was the most performant one, each mg of BiOI catalyst could degrade 40 µg Rhodamine B after three hours under visible light irradiation, while sample CP_i-Pr_s.c. was only the second most performant one in visible light and the third one in UV. However, it had the highest reaction rate (9.2 × 10^−3^ min^−1^ and 10.8 × 10^−3^ min^−1^, respectively). Therefore, the MQ is the most suitable suspension matrix used during the scale-up procedure (Table 3).

### 3.8. Outlook for Scalability

Considering the results mentioned earlier, the size of the reactor, including the paper’s size was scaled-up. Considering the findings, water was chosen as the catalyst dispersion liquid phase, and the spray-coating procedure was repeated five times in order to increase the surface coverage. The dimension of the ceramic paper sheet was 0.5 × 1 m. After the immobilization procedure, the BiOI layer was investigated by the same methods as used in the the lab-scale papers. The crystal structure, the morphology, and the optical properties were unchanged, only the amount of BiOI increased, and the coverage reached 100% (Figure S4). The pilot industrial photocatalytic system was scaled-up as well; after that the successfulness of the immobilization and the durability of the BiOI layer were investigated with a simulated photocatalytic test. Am amount of 50 L (Figure S5) of water was circulated, while the uniform BiOI layer integrity was monitored. After 3 h visible changes were not observed on the BiOI layer. This test demonstrated that the reactor construction and the spray-coating immobilization technique is potentially applicable on an industrial scale and could open the way to use this system as real water treatment equipment in the future.

## 4. Discussion of the BiOI Photocatalysts Uniform Layer Formation

The immobilized BiOI proved to be durable, photocatalytically active, and stable. This is due to the developed brand-new spray-coating technique at room temperature. For the anchoring process, the following suggestion can be made:

After the spraying step, the coated ceramic paper turned black, as already mentioned in the experimental section. This black material was most probably BiI_3_ (Figure S6), which formed during the solvent evaporation when the precipitated bismuth nitrate reacted with the dissolved potassium iodide. The remaining KI and bismuth salt were washed off the ceramic paper. After the evaporation and stabilization step, the BiOI was crystalized by the BiI_3_ direct hydrolysis during the purification step [29,57]. It means that the I_2_ was released into the solution phase, which was proven by the so-called starch test, in which amylose and I_2_, together with I¯ forms a complex compound.

The analogue of the above-mentioned bismuth oxyiodide two-step spray-coating immobilization technique could open several opportunities for immobilizing many other visible light active photocatalysts, such as all BiOX, AgX, or Ag_3_PO_4_. Furthermore, using this technique, even more durable photocatalyst layers can be created due to the lack of additional fixing materials.

## 5. Conclusions

In this paper, a brand-new two-step BiOI immobilization technique was developed by a spray-coating technique at room temperature, for maybe the first time in the literature. The coverage of the as-prepared BiOI photocatalyst layers were improved by varying the solvent and repeating the spray-coating steps. A uniform BiOI layer was created successfully with an alcoholic solvent, which morphology was followed the characteristic BiOI morphology. The infrared results suggest that the bismuth oxyiodide may anchor to the Al-O unit directly and the epoxy resin did not participate in the formation of the BiOI layer. A clear correlation was found between the crystal structure and the optical properties: the I_(102)_/I_(110)_ crystallographic plane ratio defined the band gap of the BiOI layer. It could be attributed to the high amount of the bismuth iodide external bonds, according to the Raman measurement information. The XPS result proved that the surface iodide, more precisely the amount of the external Bi-I bindings, could define the optical properties and the band gap of the BiOI photocatalyst layer. The formed crystalline BiOI layers showed excellent photocatalytic activity under UV and visible light irradiation, which were reproducible. The spray-coating procedure was successfully scaled-up to pilot-plant scale without any significant change in the catalyst properties.

## Figures and Tables

**Table 1:** The calculated crystal size based on the Scherrer equation, the amount of immobilized BiOI on 8 × 20 cm ceramic paper sheet, and the calculated surface coverage.

Sample ID	Particle Size (nm)	Immobilized BiOI (mg)	Surface Coverage (%)
P_EG_h.t.	11	×	×
CP_EG_h.t.	12	240	90.5%
CP_MQ_s.c.	21	50	82.7%
CP_i-Pr_s.c.	11	130	70.4%
CP_EtOH_s.c.	23	180	73.7%
CP_EtOH+MQ_s.c.	21	440	99.6%
CP_EtOH+EG_s.c.	21	80	59.3%

**Table 2:** The atomic ratio of the Bi and I on the BiOI coated ceramic papers, measured by SEM–EDX. The ratios of the iodine to bismuth were approximately one to one.

Sample ID	Iodine (atomic %)	Bismuth (atomic %)
P_EG_h.t.	12.50	14.49
CP_EG_h.t.	2.67	2.86
CP_MQ_s.c.	0.78	0.84
CP_i-Pr_s.c.	1.83	1.76
CP_EtOH_s.c.	4.03	4.78
CP_EtOH+MQ_s.c.	3.63	4.17
CP_EtOH+EG_s.c.	3.23	3.21

**Table 3:** Different approaches to the photocatalytic activity. The reaction rate approached the activity from the RhB site, while the mass normalized activity approached from the BiOI site.

Sample Name	Reaction Rate k_UV_ (min^−1^)	Reaction Rate k_Vis_ (min^−1^)	UV Degraded RhB/BiOI (µg/mg)	Vis Degraded RhB/BiOI (µg/mg)
CP_EG_h.t.	1.0 × 10^−3^	9.2 × 10^−3^	11	17
CP_MQ_s.c.	7.9 × 10^−3^	4.7 × 10^−3^	34	40
CP_i-Pr_s.c.	9.2 × 10^−3^	10.8 × 10^−3^	13	21
CP_EtOH_s.c.	8.0 × 10^−3^	7.4 × 10^−3^	9	13
CP_EtOH+MQ_s.c.	6.1 × 10^−3^	7.6 × 10^−3^	5	6
CP_EtOH+EG_s.c.	7.2 × 10^−3^	3.2 × 10^−3^	26	17

## Supplementary Materials

The following are available online at https://www.mdpi.com/2079-4991/10/10/1959/s1, Figure S1: Crystallographic- (a), optical- (b) and morphological (c, d) properties of the bulk ceramic paper.; Figure S2: Deconvoluted Raman spectra of CP_EtOH+EG_s.c and identify the overlapped B_1g_ external Bi-I bond and the A_1g_ external bond.; Figure S3: Kinetic linear simulation lines of RhB over the BiOI coated papers.; Figure S4: Scaling-up immobilization procedure (a), the caused changes on the XRD patter (b), and SEM micrographs after the 5^th^ spray-coating process (c, d).; Figure S5: Durability test with the scaled-up pilot bath reactor with 0.5 m^2^ BiOI coated ceramic paper and 50 L RhB contaminated water as simulated photocatalytic test. Figure S6: XRD pattern from the BiI_3_ layer on the ceramic paper.
