# Supplementary material for: Innovative and Cost-Efficient BiOI Immobilization Technique on Ceramic Paper—Total Coverage and High Photocatalytic Activity
Source: Nanomaterials (Basel). 2020 Oct 1;10(10):1959. doi: 10.3390/nano10101959 (PMC7599943; doi:10.3390/nano10101959)
Supplement: Supplementary file 1 [file nanomaterials-10-01959-s001.pdf]

nanomaterials

Supporting Information

Innovative and Cost-Efficient BiOI Immobilization Technique on Ceramic Paper-Total Coverage and High Photocatalytic Activity

Zsolt Kása [1,*], Eszter Orbán [2], Zsolt Pap [3,4], Imre Ábrahám [5], Klára Magyari [3,4], Seema Garg [6] and Klara Hernadi [1,*]

1 Department of Applied and Environmental Chemistry, University of Szeged, 6720 Szeged, Rerrich Béla sqr. 1, 6720, Szeged, Hungary
2 Department of Organic Chemistry, University of Szeged, Dóm sqr. 8, 6720, Szeged, Hungary; eszterorban94@chem.u-szeged.hu
3 Institute of Environmental Science and Technology, University of Szeged, Tisza Lajos blvd. 103, 6720, Szeged, Hungary; pzsolt@chem.u-szeged.hu (Z.P.); klara.magyari@ubbcluj.ro (K.M.)
4 Nanostructured Materials and Bio-Nano-Interfaces Center Interdisciplinary Research Institute on Bio-Nano-Sciences, Babes-Bolyai University, Treboniu Laurian Str. 42, 400271, Cluj-Napoca, Romania
5 UniChem Ltd., T. 491, Kistelek, 6760, HUNGARY; abrahamimre@unichem.hu
6 Amity Institute of Applied Sciences, Amity University, Sector 125, Noida, Uttar Pradesh, 201313, India; sgarg2@amity.edu
* Correspondence: kasa.zsolt@chem.u-szeged.hu (Zs.K.); hernadi@chem.u-szeged.hu (K.H.); Tel.: +36304286022 (Zs.K.)

Received: 26 August 2020; Accepted: 29 September 2020; Published: date

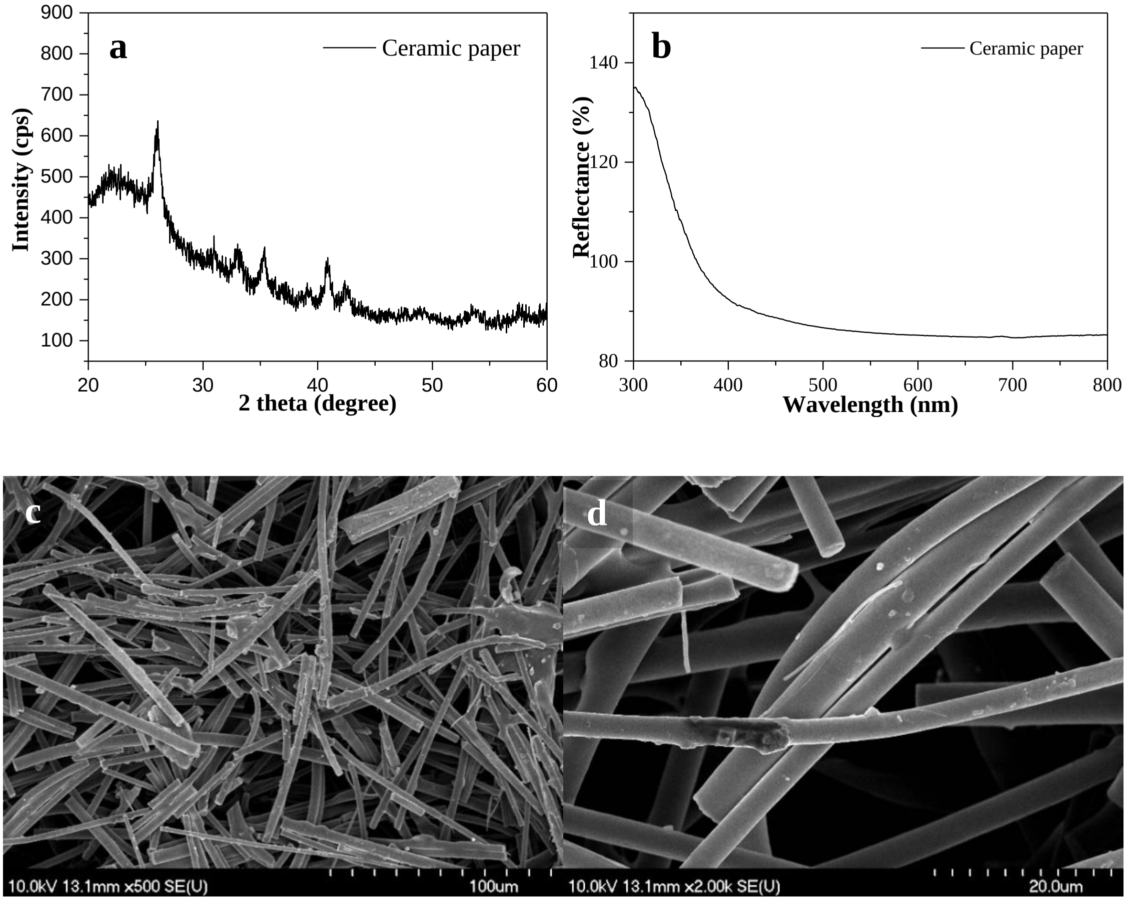

Figure S1. Crystallographic- (**a**), optical- (**b**) and morphological (**c**, **d**) properties of the.

bulk ceramic paper.

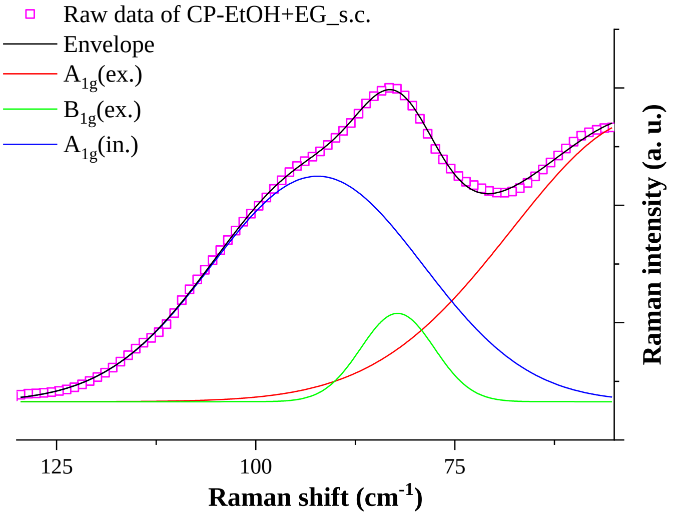

Figure S2. Deconvoluted Raman spectra of CP_EtOH+EG_s.c and identify the overlapped B_{1g} external Bi-I bond and the A_{1g} external bond.

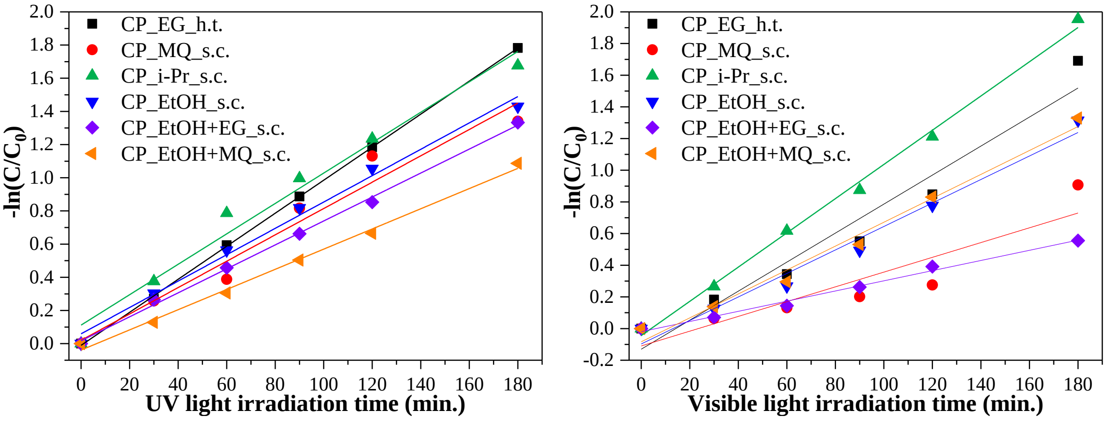

Figure S3. Kinetic linear simulation lines of RhB over the BiOI coated papers.

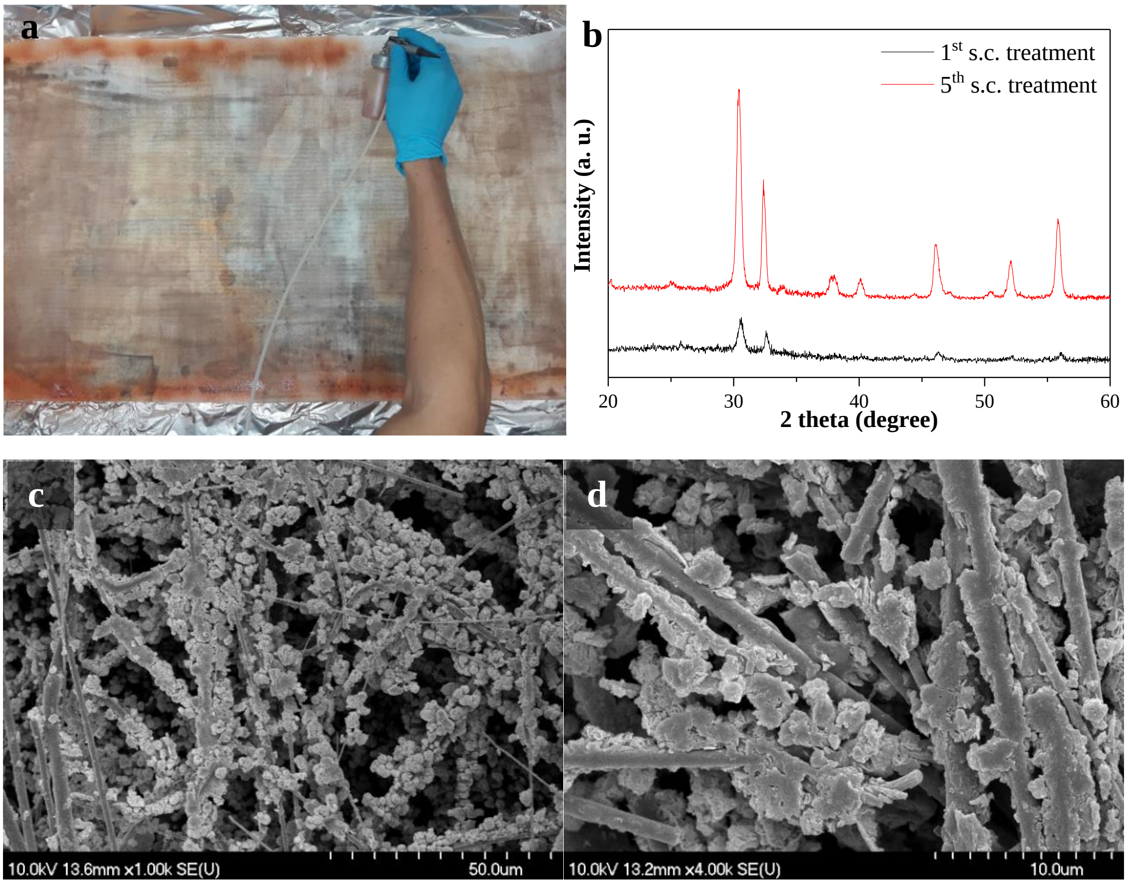

Figure S4. Scaling-up immobilization procedure (**a**), the caused changes on the XRD patter (**b**), and SEM micrographs after the 5th spray-coating process (**c**, **d**).

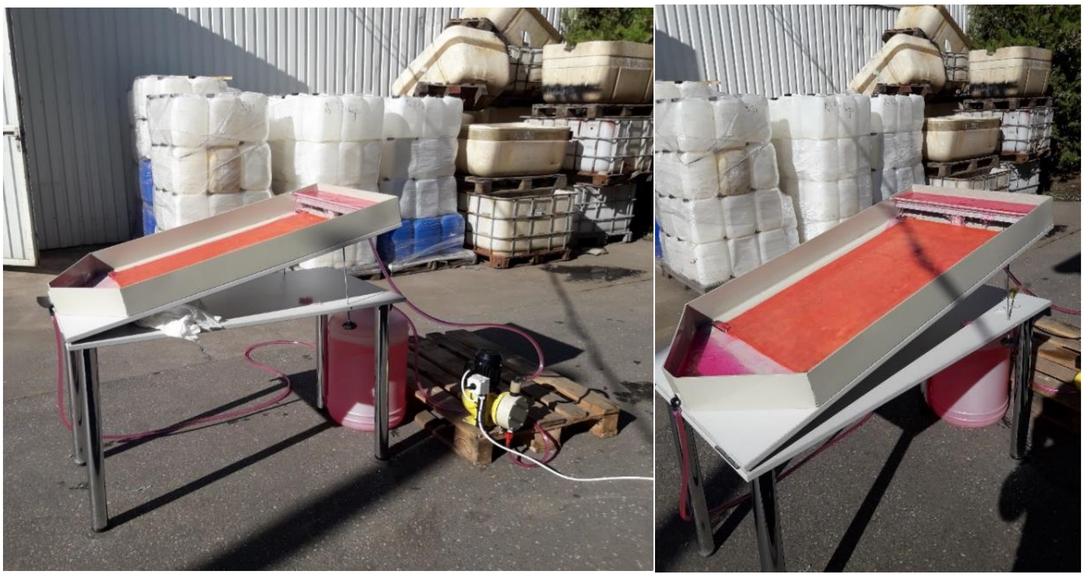

Figure S5. Durability test with the scaled-up pilot bath reactor with 0.5 m[2] BiOI coated ceramic paper and 50 LRhB contaminated water as simulated photocatalytic test.

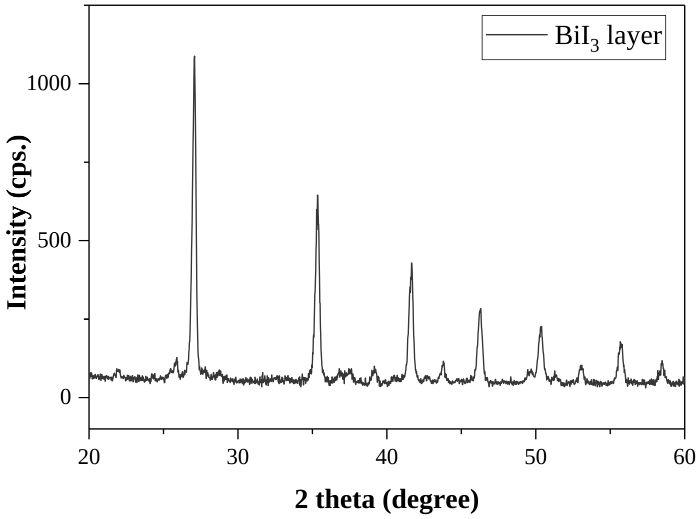

Figure S6. XRD pattern from the BiI_3 layer on the ceramic paper
